# Supplementary material for: Organo-Montmorillonite Modified by Gemini Quaternary Ammonium Surfactants with Different Counterions for Adsorption toward Phenol
Source: Molecules. 2023 Feb 21;28(5):2021. doi: 10.3390/molecules28052021 (PMC10004245; doi:10.3390/molecules28052021)
Supplement: Supplementary file 1 [file molecules-28-02021-s001.zip › molecules-2169760-supplementary.pdf]

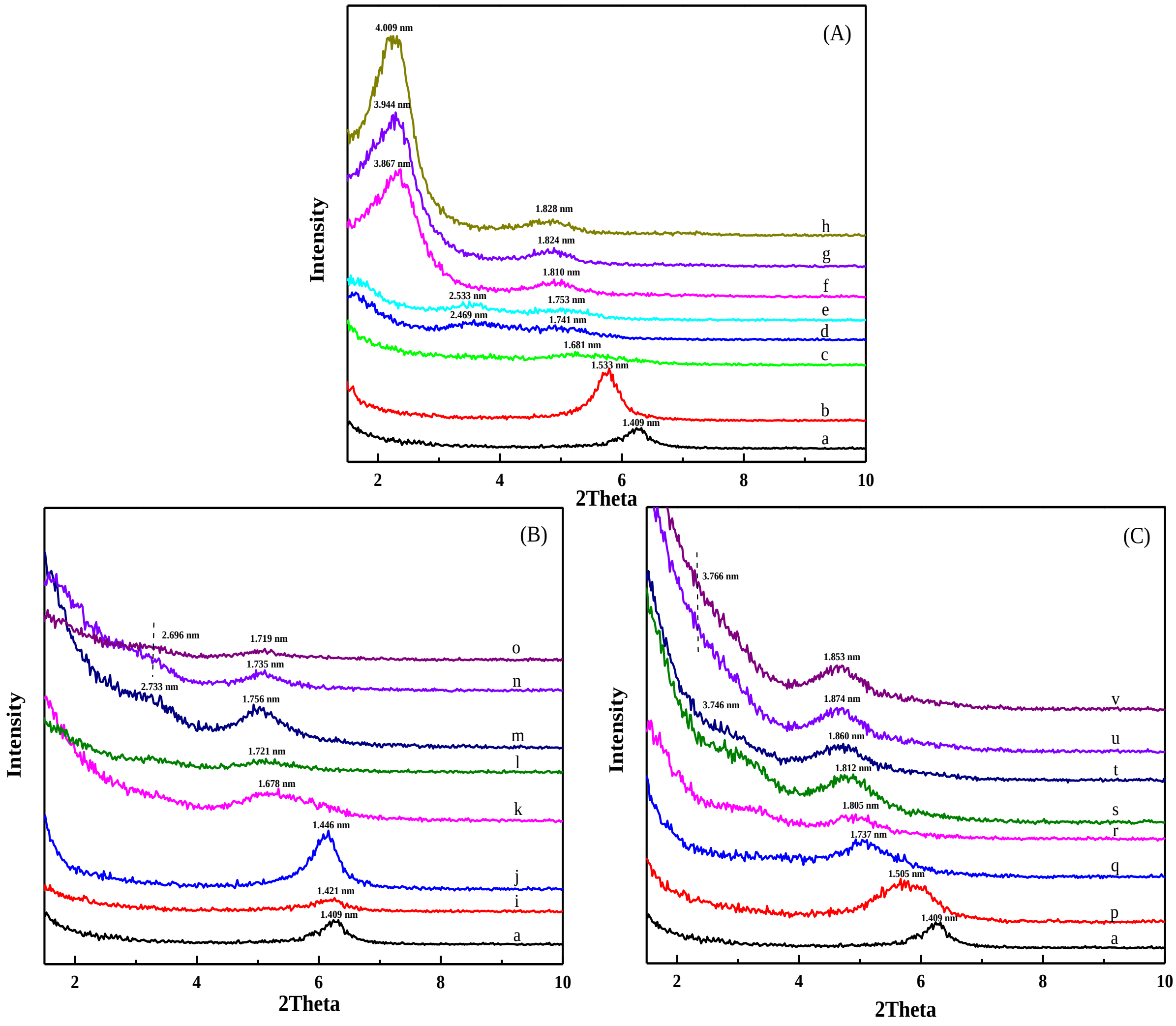

Figure S1. XRD patterns of Na-Mt(a), and MMt-12-2-12·2$CH_3CO_3^-$ (A), MMt-12-2-12·2$C_6H_5COO^-$ (B), MMt-12-2-12·2Br^-(C) under different CEC [0.4 CEC (b, i, p) ,0.8 CEC (c, j, q),1.2 CEC (d, k, r),1.6 CEC (e, l, s), 2.0 CEC (f, m, t), 2.4 CEC (g, n, u), 2.8 CEC (h, o, v)] modification conditions

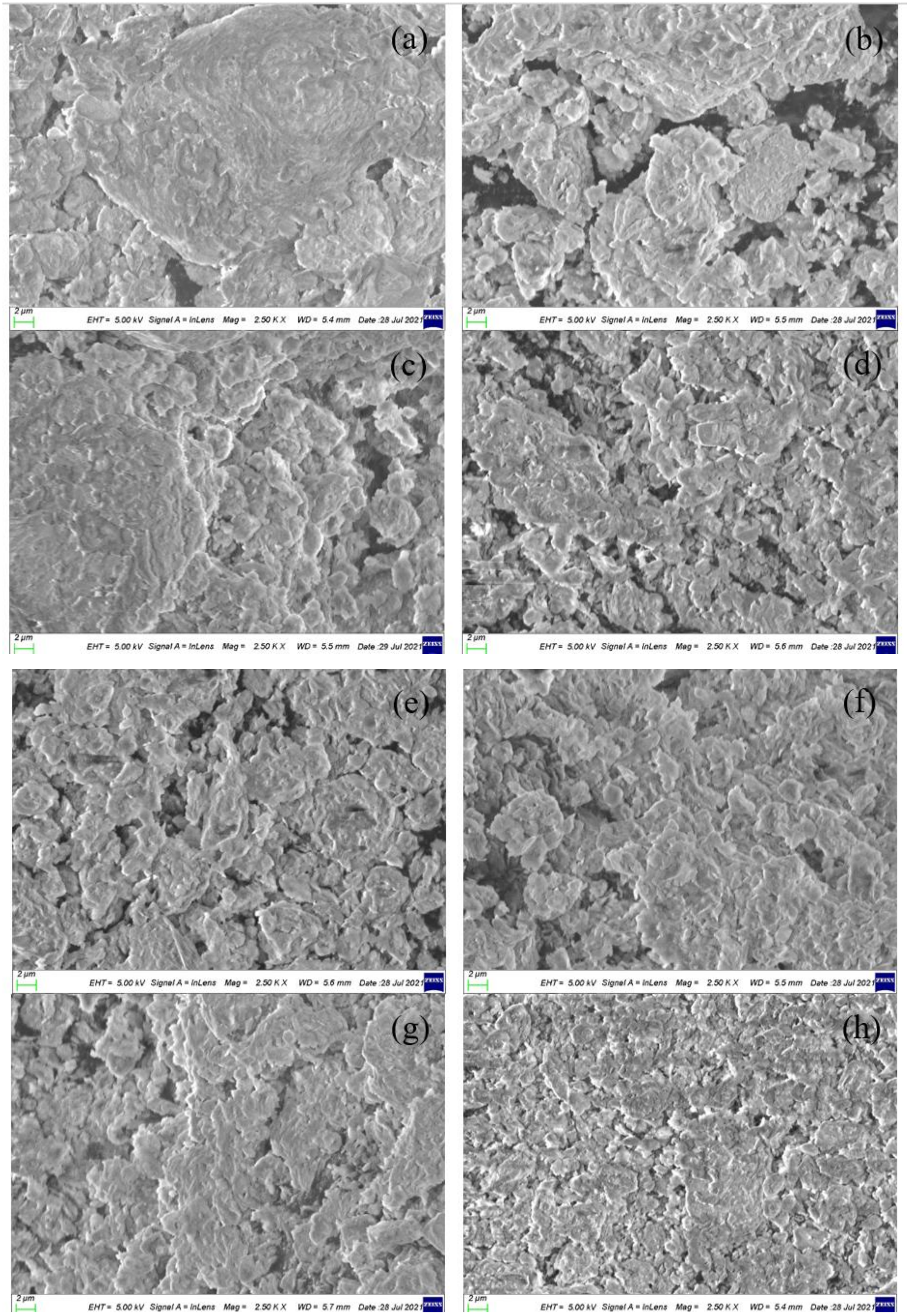

Figure S2. SEM images of Na-Mt (a), and MMt-12-2-12·2$CH_3CO_3^-$ under different CEC modification conditions (b: 0.4 CEC, c: 0.8 CEC, d: 1.2 CEC, e:1.6 CEC, f: 2.0 CEC, g: 2.4 CEC, h: 2.8 CEC)

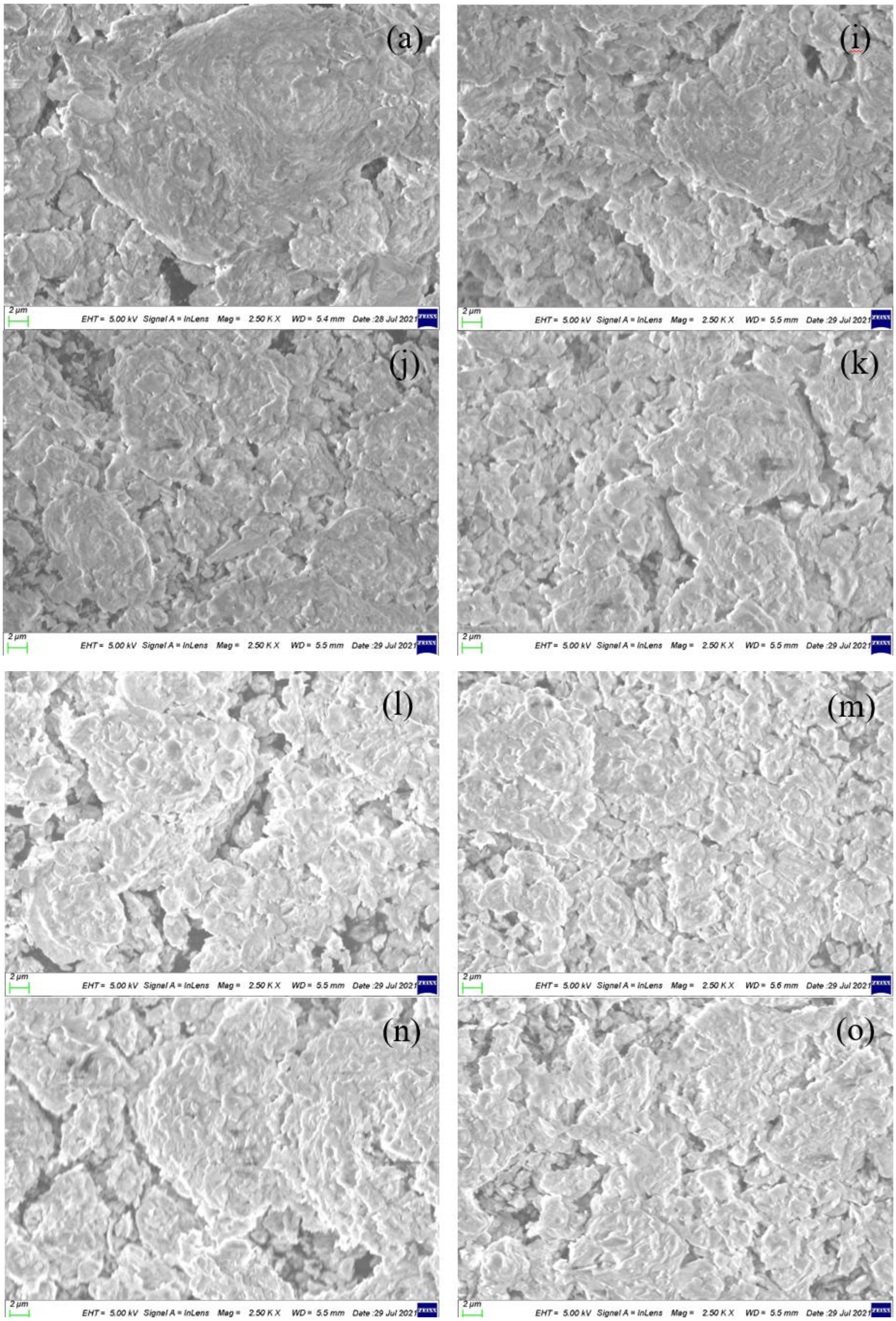

Figure S3. SEM images of Na-Mt (a), and MMt-12-2-12·$2C_6H_5COO^-$ under different CEC modification conditions (i: 0.4 CEC, j: 0.8 CEC, k: 1.2 CEC, l:1.6 CEC, m: 2.0 CEC, n: 2.4 CEC, o: 2.8 CEC)

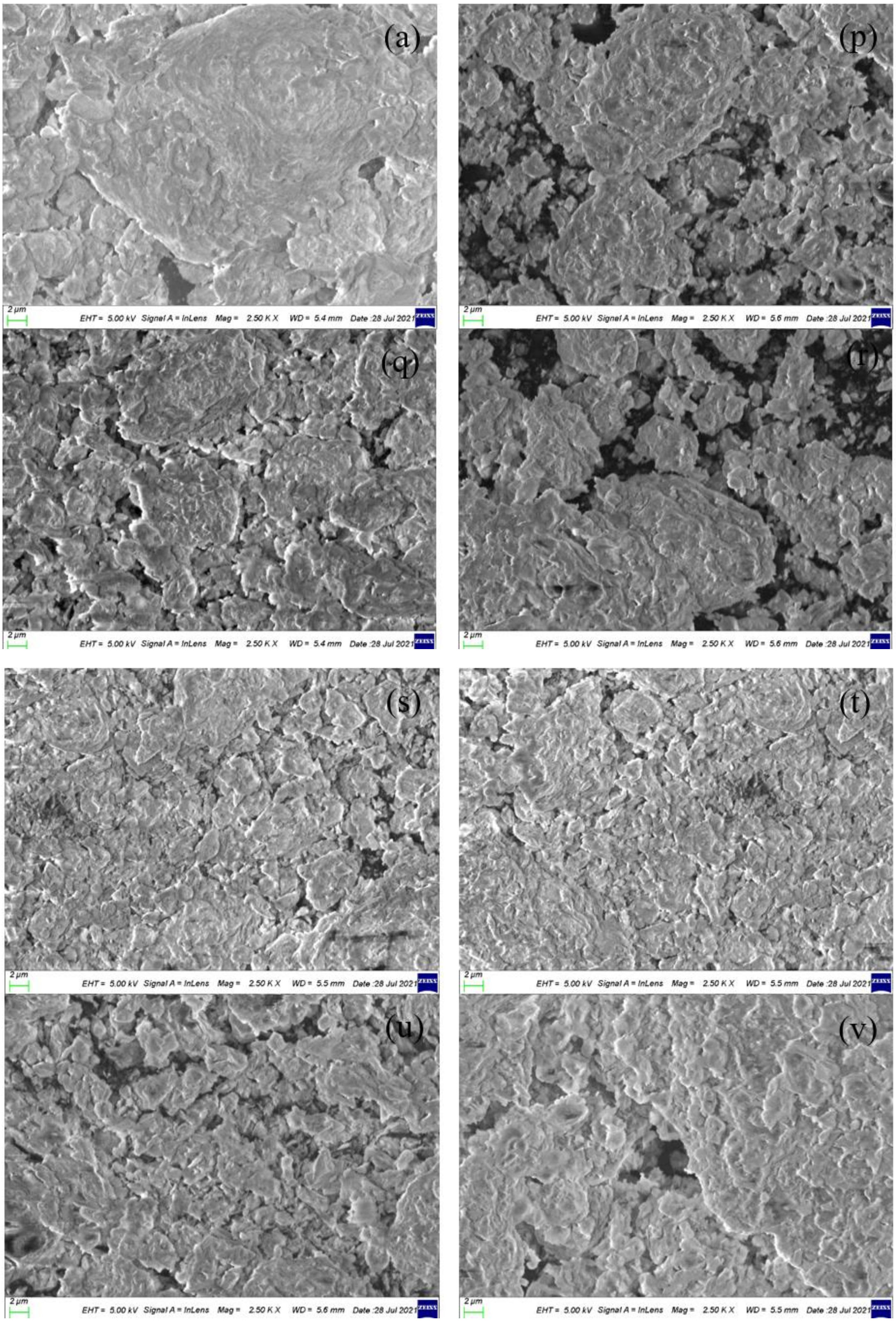

Figure S4. SEM images of Na-Mt (a), and MMt-12-2-12·$2Br^-$ under different CEC modification conditions (p: 0.4 CEC, q: 0.8 CEC, r: 1.2 CEC, s:1.6 CEC, t: 2.0 CEC, u: 2.4 CEC, v: 2.8 CEC)
